# Practical Notes on Diagnosis and Treatment

**Published:** 1906-11-17

**Authors:** 


### Practical Notes on Diagnosis and Treatment.

**Eczema of the Genitals.**

This condition, especially in the female, loudly calls for an examination of the urine for sugar, even though other symptoms of diabetes be entirely absent.

**Submucous Turbinectomy.**

Mr. Stuart-Low advocates submucous re-section of the inferior or middle turbinated bones in cases of nasal obstruction. In this way whilst the obstruction is removed there is no loss of the erectile tissue or mucous membrane. Hence the value of these structures in warming, filtering, cleansing and moistening the respired air is preserved.

**Acupuncture in Lumbago.**

In the acute stage acupuncture often gives prompt relief, and this more particularly in severe and sudden cases. The needles must be inserted on each side of the spine to a depth of two inches or more. Each should be left *in situ* for two to three minutes. Afterwards a fomentation may be applied, and in the course of half an hour or so the patient should be encouraged to take some walking exercise.

**Delayed Union in Fractures.**

In many cases of delayed union the question is largely one of feeding the patient. You must take care to feed him on something which is likely to make bone, and there is nothing more useful than taking milk to the extent of some pints, or even quarts, daily.—*Mr. Bowlby.*

**Oil of Cinnamon in Influenza.**

Dr. Joseph Carne Ross, as the result of a large experience, advises oil of Ceylon cinnamon-bark in influenza. He claims that the results are not only beneficial, but prompt. The method of administration is as follows: Twelve drops every hour for two hours, then ten drops every two hours until the temperature is normal. Afterwards ten drops thrice daily for a few days. Each dose should be taken in a wineglassful of water.

**Opium in Perforated Gastric and Duodenal Ulcer.**

Opium is a dangerous drug to give for the relief of early symptoms in these conditions. Of 22 cases in which opium was *not* given 17 recovered and only 5 died. In 12 cases in which opium was given 10 died and only 2 recovered. . . . The chief factor in prognosis is the early period when operation has been undertaken.—*Mr. Alexander Miles.*

**Recrudescence of Phthisis Pulmonalis.**

Two of the greatest risks to the patient in whom phthisis pulmonalis has been arrested are over-strain and great fatigue; these appear to have a special influence in bringing on a recrudescence.—*Dr. M. S. Paterson.*

**Hetraline.**

Hetraline is of value as a urinary disinfectant. It is chiefly indicated in cystitis, acute or chronic, especially if the urine is very turbid and with a copious sediment. It is readily soluble in hot water, and can be given in doses of 5 to 7½ grains, thrice daily.

**Tachycardia.**

Dr. D. MacMillan describes the treatment adopted by a man who suffered from recurring attacks of tachycardia. The patient, rising from his bed with a pulse numbering 175 per minute, dashed cold water freely over his chest for about a minute. He then dried himself and returned to bed. In the course of ten minutes the pulse had fallen to 100, and soon after it numbered 72.

**Treatment of Exophthalmic Goitre.**

In a clinical study of 32 consecutive cases of Graves's disease Professor Dock states that he found thyroid extract to produce but little effect, and whilst thyroidectin was regarded by some patients as beneficial, it did not slow the pulse. Adrenalin, thyroid extract, and *x*-rays were all tried on some of the patients, but with negative results. Professor Dock does not put much faith in sodium phosphate nor in electricity. He relies on symptomatic treatment conducted on traditional lines.

**Massage of the Heart in Chloroform Syncope.**

Lenormant, of Paris, claims massage of the heart to be a safe and easy proceeding in desperate cases of chloroform syncope. He advises the abdominal route, and is opposed to division of the diaphragm. The ventricles should be compressed regularly and rhythmically at a rate of thirty to sixty times per minute.

**Raynaud's Disease.**

The sense of heat and itching occurring in the fingers, etc., in cases of Raynaud's disease can often be relieved by the application of an ointment of ichthyol; strength, 30 per cent.

**Scrofulous Eczema and Enlarged Glands.**

In these conditions iodipin administered internally has recently been recommended. To an infant twenty drops of a 10 per cent. solution may be given twice daily, and larger doses to older children. A careful look-out for symptoms of iodism should be kept.

**Sciatica.**

Dr. Brindley James writes that he has cured a number of cases of sciatica by the direct injection into the nerve of 15, increased, if necessary, to 30 minims of sulphuric ether. He uses a hypodermic syringe provided with a long needle.

**Digitalis in Aortic Disease.**

"Although digitalis is the most powerful remedy which we possess in mitral disease, in cases of pure aortic regurgitation digitalis is not only a dangerous drug, but actually a poison."—*Dr. Seymour Taylor.*

**Rhubarb Mixture.**

The old-fashioned rhubarb mixture, consisting of rhubarb, soda, and peppermint, is an excellent combination taken after food for what we call the chronic, middle-aged, constipated, flatulent dyspeptic. Of the drugs in common use in stomach disorders laxatives occupy the first place in my estimation, and of them I believe rhubarb is the best.—*Professor Alfred G. Barrs.*

